# The Roles of Notch3 on the Cell Proliferation and Apoptosis Induced by CHIR99021 in NSCLC Cell Lines: A Functional Link between Wnt and Notch Signaling Pathways

**DOI:** 10.1371/journal.pone.0084659

**Published:** 2013-12-18

**Authors:** Chunyan Li, Siyang Zhang, Yao Lu, Ying Zhang, Enhua Wang, Zeshi Cui

**Affiliations:** 1 Center of the Laboratory Technology and Experimental Medicine, China Medical University, Shenyang, People’s Republic of China; 2 Department of Pathology, China Medical University, Shenyang, People’s Republic of China; H. Lee Moffitt Cancer Center & Research Institute, United States of America

## Abstract

Wnt and Notch signaling pathways both play essential roles and interact closely in development and carcinogenesis, but their interaction in non-small-cell lung cancer (NSCLC) is poorly unknown. Here we investigated the effects of CHIR99021, a Wnt signaling agonist, or Notch3-shRNA, or the combined application of CHIR99021 and Notch3-shRNA on cell proliferation and apoptosis, as well as the expressions of Notch3, its downstream genes, cyclinA and caspase-3. Our results showed that CHIR99021 up-regulated the expression of Notch3 protein and HES1 and HEYL mRNA. CHIR99021 promoted cell proliferation and the expression of cyclinA, which were inhibited by Notch3-shRNA in these three cell lines. Moreover, Notch3-shRNA significantly attenuated the positive effects of CHIR99021 on cell proliferation and cyclinA in H460 and H157. As for apoptosis, Notch3-shRNA induced cell apoptosis and increased the expression of caspase-3, whereas CHIR99021 showed the different effects in these three cell lines. The inhibitory effect of CHIR99021 on apoptosis was significantly weakened by Notch3-shRNA only in H460. Overall, although the effects of CHIR99021 and the combined application of CHIR99021 and Notch3-shRNA on the cell proliferation and apoptosis aren’t completely similar in the three cell lines, our findings still indicate that Notch3 signaling can be activated by canonical Wnt signaling and a functional link between Wnt and Notch signaling pathways exists in NSCLC, at least, which partially is associated with their regulations on the expressions of cyclinA and caspase-3.

## Introduction

Notch and Wnt signaling pathways are both high-conserved pathways in the development of invertebrate and vertebrate, which participate in a variety of cell biological properties including cell polarity, proliferation, differentiation, and migration. The aberrant activation of these pathways plays an important role in tumor development and progression. Moreover, the close relationship between the two signaling processes has been increasingly observed in some tumor types [1,2].

Lung cancer is the most common cause of cancer death in the world. Non-small-cell lung cancer (NSCLC) is the major classification of lung cancer, which accounts for approximately 85% of all lung cancer cases. NSCLC also appears to actively utilize the above conserved developmental pathways. Both down-regulations of the Wnt antagonists and up-regulations of the key Wnt components have been observed in NSCLC tissues, and are associated with a poor prognosis and recurrence [3-6]. The application of various molecules that target the Wnt/β-catenin signaling pathway led to the inhibition of NSCLC cell proliferation [7,8]. Notch receptors, Notch1-4, with which five ligands are capable of binding, have been identified in mammals. Compared with the Wnt signaling pathway, studies on Notch signaling pathway in NSCLC remain small in number. Previous studies concerned with the pathogenesis of lung cancer have identified that Notch3 played an essential role in NSCLC [9-13]. However, regarding to the cross talk between the two signaling pathways in NSCLC, the data are not much.

In our previous study, we found that lithium chloride (LiCl), which inhibited glycogen synthase kinase 3β (GSK-3β) activity in vivo and vitro, and mimicked the biochemical effect of Wnt signaling by leading to stabilization of β-catenin protein, up-regulated Notch3 signaling, and Notch3 siRNA weakened the effects of LiCl on the cell cycle of NSCLC cell lines [14]. These findings indicate that there is a potential link between the regulations of two signaling pathways on cell cycle. To further elucidate whether a functional cross talk exists between the two pathways in NSCLC, we examined the impact of CHIR99021, a Wnt signaling agonist, or Notch3-shRNA, or the combined application of CHIR99021 and Notch3-shRNA on cell proliferation and apoptosis.

## Materials and Methods

### Cell culture and treatment

Three human lung cancer cell lines (adenocarcinoma, A549; squamous cell carcinoma NCI-H157; large cell carcinoma, NCI-H460), which purchased from ATCC (U.S.A), were cultured in DMEM/F12 medium (Hyclone, P.R.C) supplemented with 10% fetal calf serum (FCS, Gibco/BRL, U.S.A). CHIR99021 (BioVision, U.S.A) was prepared at 100 µM and stored at -20°C. The cells were switched with fresh medium plus 5µM CHIR99021, or DMSO (Sigma, U.S.A) as control and were cultivated for 24 h before cell analysis.

### Immunofluorescence

The cells seeded in 6-well plate were stained according to the flowing protocol. Discard the medium mixture, add 4% paraformaldehyde to fix cells at room temperature for 10 min, wash cells with PBS for 5 min in a shaker, add 0.2% Trion X-100 for 10 min, wash cells with PBS for two times, add β-catenin(05-665, Millipore, U.S.A) or Notch3 monoclonal antibody (D11B8,Cell signaling technology, U.S.A) at 4°C overnight, wash cells with PBS containing 0.02% Triton X-100 for three times, add goat anti mouse or anti rabbit IgG labeled by FITC (1:1000, Zhongshan Bio., P.R.C) for 60 min at room temperature, wash cells with PBS containing 0.02% Triton X-100 for three times, counterstain nuclei with Hoechst33342 (5 µg/ml) for 30 min at room temperature, wash cells with PBS containing 0.02% Triton X-100 for three times, and finally, add 200 µl PBS. Images were taken using Inverted Fluorescence Microscope with Digital CCD Imaging System (IX71/DP70, Olympus, Japan).

### Notch3 short hairpin RNA (shRNA) transfection

The plasmid encoding shRNA targeting Notch3 gene (sc-37136-SH, Santa Cruz biotechnology, U.S.A) and the non-target shRNA control plasmid were transfected into three lung cancer cell lines with TranSmarter (Abmart, P.R.C.). The cells were seeded into 10 cm plates 48 h after transfection and were cultured in the medium with puromycin (Sigma-Aldrich, U.S.A) at a final concentration of 1 μg/ml for 2 weeks. Then the cell clones were picked and put into a 3.5cm plate. Then the cells were subsequently cultured with puromycin and the medium was replaced every other day. Validation of Notch3 silencing in the transfected cells was carried out by western blot analysis. The flowing experiments were performed using the cells with Notch3-shRNA.

### Quantitative real-time reverse transcription (RT)-polymerase chain reaction (PCR)

Total RNA was prepared from cultured cells using the Trizol reagent (Invitrogen, U.S.A) according to the manufacturer’s instructions. cDNA was synthesized using a kit from Takara with oligo-dT as a primer. Primers were listed in Table 1. For qPCR, reactions were run on a real-time PCR system (LightCycler 480，Roche, Switzerland/German). Gene expression was detected with SYBR Green (TaKaRa, Japan), and relative gene expression was determined by normalizing to reference gene glyceraldehyde phosphate dehydrogenase (GAPDH) using the relative quantitative 2^-ΔΔCt^ method.

**Table 1:** Oligonucleotide primers used for RT–PCR analyses.

Gene	Primer sequence (strand)	Product size (bp)
HES1	5’- ctctcttccctccggactct-3’ (+)	186
	5’- aggcgcaatccaatatgaac-3’ (-)	
HEYL	5’- caagcatgcaactccaaaga-3’ (+)	184
	5’- aggaaggcttggggatagaa-3’ (-)	
cyclinA	5’-cggcagccagcctattcttt-3’ (+)	124
	5’-gcagagttgcccaacatcac -3’ (-)	
caspase 3	5’-tgtgaggcggttgtagaagt-3’ (+)	124
	5’-tccagagtccattgattcgct-3’ (-)	
GAPDH	5’-tctgcccggagcctccttcc-3’ (+)	196
	5’-gatgcacccgctgcgcacta-3’ (-)	

### Cell cycle analysis

Cells were collected and washed with PBS, then fixed by incubating in 75% alcohol for overnight at -20°C. After washing with cold PBS three times, cells were resuspended in 1 ml PBS containing 40 μg propidium iodide (PI, Sigma, U.S.A) and 100 μg RNase A (Sigma, U.S.A), and incubated at 4°C for at least 30 min. Samples were analyzed for DNA contents using flow cytometer of FACSCalibur and ModFit software (BD Biosciences, U.S.A). Each experiment was repeated for at least three times.

### 5-Ethynyl-2’-deoxyuridine (EdU) assay

EdU incorporation assay was performed using Cell-Light™ EdU Apollo®567 In Vitro Flow Cytometry Kit (Guangzhou Ruibo Bio., P.R.C) according to the following protocol. Cells were cultured in the medium with 10μM EdU for 2h, collected using 0.25% trypsin, washed with cold PBS containing 1% BSA, resuspended in 500μl of 1×Apollo reaction buffer, and incubated at room temperature for 30 min. Then cells were washed with PBS containing 0.5% Triton X-100, stained with 1×Hoechst33342 reaction buffer for 5 min at room temperature, washed with PBS containing 0.5% Triton X-100, and finally 500 µl PBS was added. Cells were analyzed using flow cytometer of FACSAria (BD Biosciences, U.S.A). The excitation and emission of Apollo®567 are 550nm and 565nm, respectively, which are similar to the excitation and emission of allophycocyanin fluorescein(APC). Finally, samples were analyzed by FACSDiva software (BD Biosciences, U.S.A). 

### Apoptosis assay

Cell apoptosis was detected using an Annexin V-FITC apoptosis detection kit (Beyotime institute of biotechnology, P.R.C) according to the manufacturer's protocol. The cells were seeded in 6-well plates at approximately 5 × 10^5^ cells/ml. The cells were treated with CHIR99021 and incubated for 24 h. Subsequently, the cells were harvested, washed with PBS, resuspended in Annexin binding buffer, and then analyzed by FACSAria (BD Biosciences, U.S.A). Four quartos in the scatter plot of FACS represent 4 cell types, the necrotic or damaged dead cells(Q1), the late apoptosis cells (Q2), the live cells (Q3) and the early apoptosis cells(Q4), respectively. 

### Western blot analysis

Cells were lysed in phospho-lysis buffer (50 mM Tris–Cl, pH 7.5, 150 mM NaCl, 1 mM MgCl_2_, 0.5% NP40, 1 mg/ml BSA, 0.1 mM PMSF). Cell extracts were collected by centrifugation. Samples were analyzed by 10% SDS-polyacrylamide gel electrophoresis, followed by western blotting using rabbit monoclonal anti-Notch3 (D11B8, Cell signaling technology, U.S.A), rabbit polyclonal anti-cyclinA (sc-596, Santa Cruze, U.S.A), and rabbit polyclonal anti-caspase-3 (sc-98785, Santa Cruze, U.S.A), respectively, and the second antibody of goat anti-rabbit IgG conjugated with horseradish peroxidase (HRP, HuaAn Biotech, P.R.C). The membrane was developed using the chemiluminescent reagents (SuperSignal West Pico Chemiluminescent Substrate, Pierce, U.S.A). The bands were quantified by densitometry to obtain the integrated density values (IDV). The relative amounts of each protein to actin are represented as the ratio of their IDV in the histograms. 

### Statistical analysis

Data are presented as the mean ± s.d. of at least three independent experiments. Statistical analysis was performed using Student’s t test, and a value of P < 0.05 was considered to be significant. P < 0.05 is represented by *, P < 0.01 is represented by **, and no significance is represented as ns in figures.

## Results

### CHIR99021 treatment led to the accumulation of β-catenin in the cytoplasm and the nucleus

**Figure 1:**
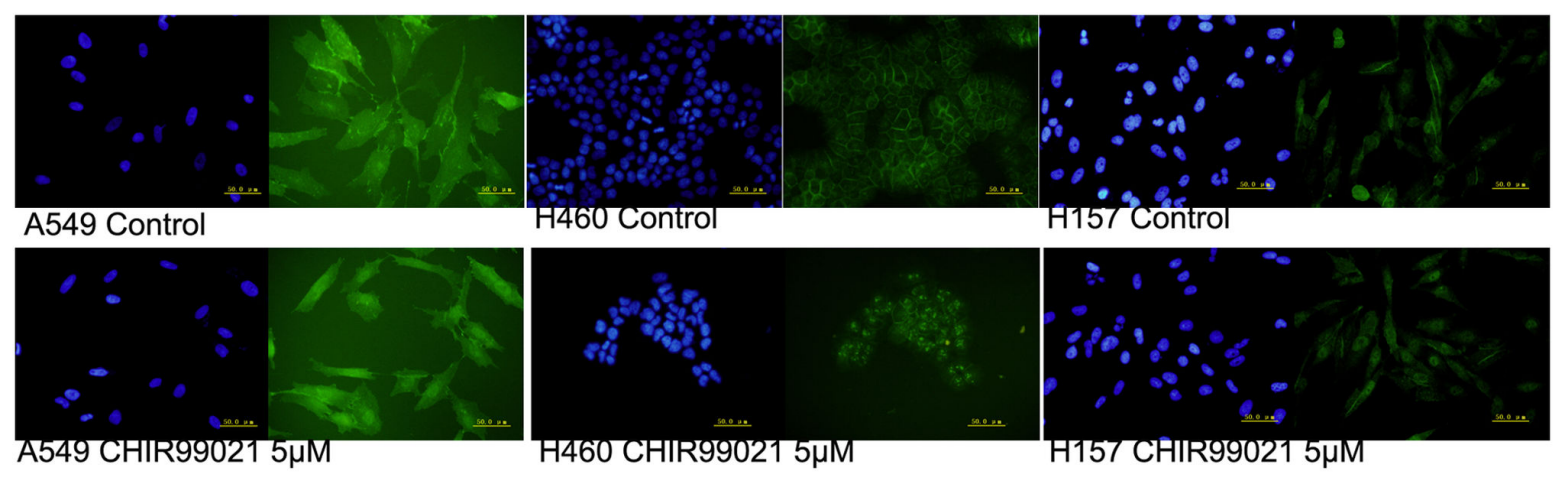
CHIR99021 treatment led to the accumulation of β-catenin in the cytoplasm and the nucleus. Untreated and treated with CHIR99021 for 24h, the cells were analyzed by immunofluorescence staining with a monoclonal anti-β-catenin (green in cytoplasm and nuclei). Nuclei were counterstained with Hoechst33342 (blue). Scale bars are 50μm.

The content and distribution of β-catenin, a major component of canonical Wnt signaling, play a key role in the activation of canonical Wnt signaling. The inhibition to GSK-3β leads to β-catenin accumulation in cytoplasm and translocation into the nucleus where it interacts with T-cell factor (TCF)/lymphoid enhancer factor (LEF) and activates transcription of downstream target genes. CHIR99021 is a potent and high selective inhibitor of GSK-3β, which is used to mimic the biochemical effect of Wnt signaling [15,16]. In this study, the expression of β-catenin was mainly located in membrane and cytoplasm of control cells. Especially, the significant membrane staining of β-catenin was observed in all the three cell lines. After CHIR99021 treatment, cells were present with cytoplasmic and nuclear staining of β-catenin, and the accumulation in nuclei confirmed the translocation of β-catenin from cytoplasm to nucleus (Figure 1).Thus, the findings above demonstrate that CHIR99021 treatment may increase the content of β-catenin protein in the cytoplasm and result in the translocation of β-catenin protein into the nucleus in NSCLC cells.

### CHIR99021 up-regulated the expression of Notch3 protein and the mRNA expressions of its down-stream genes, which were specifically reversed by Notch3-shRNA.

**Figure 2:**
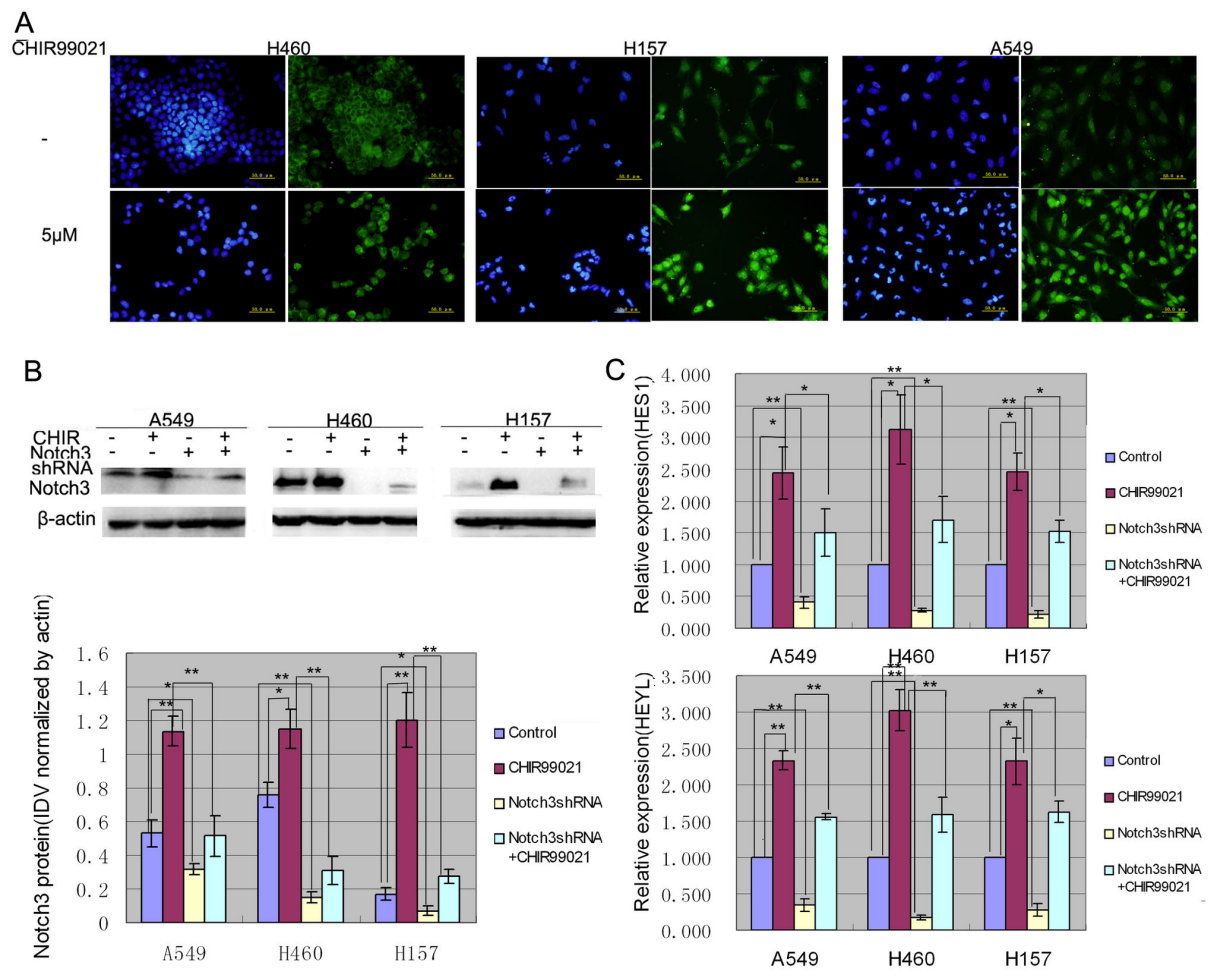
CHIR99021 up-regulated the expression of Notch3 protein and the mRNA expressions of its down-stream genes, which were specifically reversed by Notch3-shRNA. (A) Untreated and treated with CHIR99021 for 24h, the cells were analyzed by immunofluorescence staining with a monoclonal anti-Notch3 (green in cytoplasm and nuclei). Nuclei were counterstained with Hoechst33342 (blue). Scale bars are 50μm. (B)Western blot panels represent the expression of Notch3. The densitometric analysis was performed with images of three independent experiments and the histogram of results is shown on the bottom. (C)Real-time RT-PCR was performed to evaluate the expressions of HES1 and HEYL. Error bar represents mean ± s.d..

Our previous results showed that LiCl treatment increased the expressions of Notch3 and its down-stream genes in NSCLC cell lines. To further identify the effect of Wnt signaling, the expressions of Notch3 and its down-stream genes in the cells with or without CHIR99021 treatment was examined by immunofluorescence, Western blotting and real-time RT-PCR. As shown Figure 2A, the expression of Notch3 protein was mainly located in cytoplasm and nucleus of the control cells. After exposure to CHIR99021 for 24h, cells were still present with cytoplasmic and nuclear staining of Notch3, but the staining of Notch3 in nuclei seemed to be higher than that in control. This finding suggests that CHIR99021 treatment can not only increase the content of Notch3 protein but also promote the translocation of Notch3 protein into the nucleus in NSCLC cells. Then, the results from Western blotting and real-time PCR further confirmed that CHIR99021 significantly un-regulated the expressions of Notch3 protein and its down-stream genes, HES1 and HEYL at mRNA level that was consistent with that previously reported, respectively (Figure 2B, C). Moreover, the up-regulation was significantly inhibited by specific Notch3-shRNA, which efficiently silenced Notch3 expression (Figure 2B, C). Thus, these results suggest that Notch3 signaling can be activated by canonical Wnt signaling.

### Notch3 was involved in cell proliferation induced by CHIR99021 in H460 and H157

**Figure 3:**
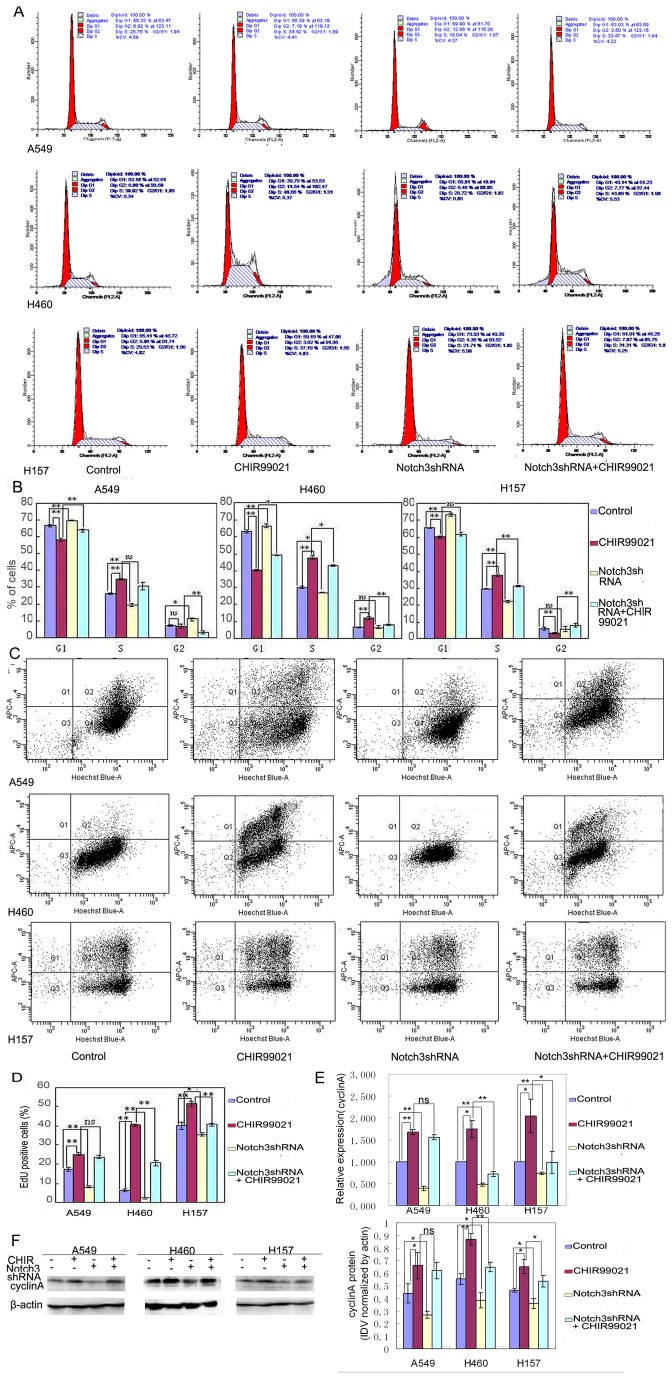
Notch3 was involved in the alteration of cell cycle and proliferation induced by CHIR99021 in H460 and H157. (A) The distribution of cells in different cycle phases was analyzed by flow cytometric analysis with or without the reduction of Notch3 in presence or absence of CHIR99021. (B) Histograms represent the percentages of different cycle phases.(C) The cells incorporated with EdU were analyzed by flow cytometric analysis with or without the reduction of Notch3 in presence or absence of CHIR99021.The cells in Q2 are the EdU positive cells. (D) Histogram represents the percentages of EdU positive cells. (E) Real-time RT-PCR was performed to evaluate the expression of cyclinA. (F) Western blot panels represent the expression of cyclinA. The histogram of densitometric analysis is shown on right. Error bar represents mean ± s.d..

The cell cycle is a tightly regulated series of events that governs cell replication and division. The dysregulation of cell cycle leading to aberrant cell proliferation is considered as the basic biological character of cancer [17]. To explore the effects of Notch3-shRNA and CHIR99021 on the proliferation of lung cancer cells，the cell cycle distribution was firstly analyzed. As shown in Figure 3A and B, CHIR99021 treatment significantly increased the percentages of S phase and decreased the percentages of G1 phase, while Notch3-shRNA increased the percentages of G1 phase and decreased the percentages of S phase, compared with control cells. In addition, the presence of Notch3-shRNA significantly weakened the effect of CHIR99021 on G1 phase in A549, on S phase in H157 and on both S and G1 phase in H460 when compared to cells only with CHIR99021 treatment. 

To further assess the effects of Notch3-shRNA and CHIR99021 on proliferation of lung cancer cells, the cells were analyzed by employing EdU incorporation assay. As shown in Figure 3C and D, the percentages of EdU positive cells in the presence of CHIR99021 were higher than that in control cells, while the cells with the reduced expression of Notch3 showed less EdU positive cells than control cells. Moreover, Notch3-shRNA efficiently attenuated the increase of EdU positive cells induced by CHIR99021 in H157and H460. Thus, data from the results of cell cycle and EdU incorporation assays in H157 and H460 suggest that Notch3 is probably involved in cell proliferation induced by CHIR99021.

As known, both Wnt and Notch pathways mediate the biological properties by the modulation of the expressions of a set of genes. Cell proliferation is controlled at specific points in the cell cycle, particularly at the G1 to S and the G2 to M transitions. Since the S-phase alteration induced by Notch3-shRNA and CHIR99021 were observed, we used Western blotting and real-time RT-PCR to examine the expression of cyclinA, an S phase-specific cell cycle regulatory protein, in order to further explore their interplay on cell proliferation. As presumed, CHIR99021 up-regulated the expression of cyclinA, while Notch3-shRNA down-regulated its expression. Moreover, Notch3-shRNA partially inhibited the increase of cyclinA induced by CHIR99021 in H157 and H460 (Figure 3E, F).These change tendencies of cyclinA protein and mRNA expressions were consistent with those of cell cycle and proliferation mentioned above. 

### Notch3-shRNA weakened the inhibitory effect of CHIR99021 on apoptosis in H460 but not in A549 and H157

**Figure 4:**
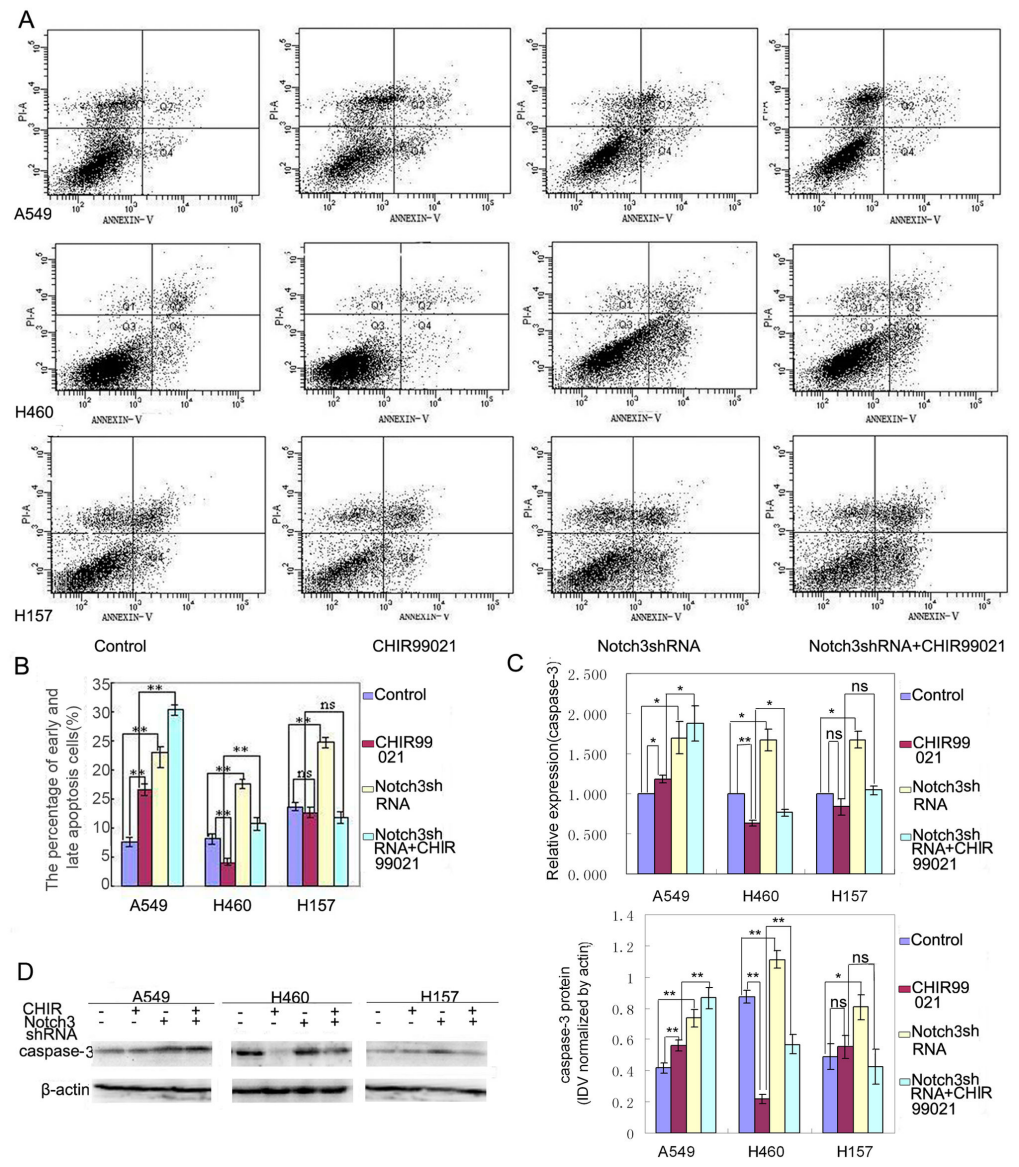
Notch3-shRNA weakened the inhibitory effect of CHIR99021 on apoptosis in H460 but not in A549 and H157. (A) Cell apoptosis was analyzed by flow cytometry. The cells in Q2 and Q4 represent late and early apoptosis cells, respectively. (B) Histogram represents the percentages of early and late apoptosis cells. (C) Real-time RT-PCR was performed to evaluate the expression of caspase-3. (D) Western blot panels represent the expression of caspase-3. The histogram of densitometric analysis is shown on right. Error bar represents mean ± s.d.

Suppression of apoptosis is considered as another trigger of carcinogenic progression [17,18]. To assess the effects of Notch3-shRNA and CHIR99021 on cell apoptosis, we examined the translocation of phosphatidylserine through cell membrane, a key apoptotic process, using an Annexin V-FITC apoptosis detection kit. Our results showed that the reduction of Notch3 expression induced the increase of cell apoptosis in all three lung cancer cell lines. However, the effects of CHIR99021 on cell apoptosis were variable. CHIR99021 treatment significantly attenuated cell apoptosis in H460, while it did not significantly affect apoptosis in H157 and significantly increased apoptosis in A549 (Figure 4A, B). Meanwhile, the reduced expression of Notch3 significantly weakened the inhibitory effect of CHIR99021 on cell apoptosis in H460, whereas it significantly synergized the positive effect of CHIR99021 on cell apoptosis in A549. Although the alterations of apoptosis induced by CHIR99021 and their combined application were different in these three cell lines, these observations above, at least, indicate that there is some interplay between Notch3-shRNA and CHIR99021 in the regulation of cell apoptosis. 

Caspase-3 is a key executioner of apoptosis [18]. To further explore the effects of Notch3-shRNA and CHIR99021 on cell apoptosis, we examined the expression of cleaved caspase-3 by Western blotting and real-time RT-PCR. As shown in Figure 4C, D, CHIR99021 considerably inhibited the expression of caspase-3 in H460 cells and Notch3-shRNA increased the expression of caspase-3 in all three lung cancer cell lines. The down-regulation of caspase-3 induced by CHIR99021 was only weakened by the reduction of Notch3 in H460. These results further suggest that the cooperative roles of Notch3 and CHIR99021 on apoptosis might exist in H460. 

## Discussion

Previous studies have shown that the activations of Wnt and Notch3 signaling pathways promoted the proliferation and survival of NSCLC cells, respectively, suggesting that both Wnt and Notch3 pathways contribute to the tumorigenicity of NSCLC and there may be some relationship between them. The cross talk between Wnt and Notch3 pathways has been observed in some tumors. In primary melanoma cells, the activation of Notch1 signaling enables them to gain metastatic capability and the oncogenic effect of Notch1 is β-catenin-dependent [19]. In Wnt-1-transformed mouse mammary epithelium cells, Notch signaling is up-regulated and is required for the presence of tumorigenic phenotype. Moreover, increased Notch signaling in primary human mammary epithelial cells is sufficient to reproduce some aspects of Wnt-induced transformation [20]. Notch and Wnt signals cooperatively control cell proliferation and tumorigenesis in the intestine [21]. Our initial experiments showed that Wnt signaling pathway up-regulated not only the expression of Notch3 but also its downstream targets HES and HEYL. Meanwhile, inhibition of Notch3 attenuated the effect of Wnt signalings on cell cycle [14]. It is widely accepted that the aberrant cell cycle is closely associated with cell proliferation and apoptosis. Thus, these data inspired us to further explore whether a cross talk between Wnt and Notch signaling pathways is utilized to regulate cell proliferation and apoptosis. In this study, we firstly confirmed that CHIR99021 up-regulated the expression of Notch3 protein and the mRNA expressions of its down-stream genes. CHIR99021 and Notch3 synergistically promoted the proliferation H460 and H157. Unlike its effect on cell proliferation, CHIR99021 showed the different effects on apoptosis in the three cell lines. CHIR99021 only significantly inhibited apoptosis in H460, which was buffered by Notch3 knockdown. As known, as an over-achieving protein kinase, inhibition of GSK-3β leads to the activation of a vast array of substrates [22], which might play different roles on cell apoptosis, in addition to β-catenin. Indeed, inhibition of GSK-3β activity has been shown to trigger apoptosis only in human pancreatic cancer cells but not in human non-transformed pancreatic epithelial cells through JNK pathway [23]. Thus, whether the regulation of CHIR99021 on apoptosis is positive or negative might depend on cell type and context. Similarly, whether the interaction between Notch3 and CHIR99021 is synergistic or antagonistic might also depend on cell type and context. Overall, our results suggest a functional link between both signaling pathways in the regulation of NSCLC progression.

In addition, we observed the regulated expressions of cyclinA and caspase-3. The observed change tendencies of cyclinA or caspase-3 expressions were consistent with those of cell proliferation and apoptosis, further supporting the possibility of a functional link between Wnt and Notch3 signaling pathways. Although no report has shown that cyclinA and caspase-3 are the direct target genes of Wnt and Notch pathways, the regulations of them by both pathways have been found. In hepatocellular carcinoma cells, the up-regulation of cyclinA has been observed after the activation of Wnt/β-catenin signaling pathway [24]. Down-regulation of Notch signaling pathway decreases the expression of cyclinA in Ishikawa endometrial cancer cells [25] and pancreatic cancer cells [26]. Inhibition of Wnt signaling pathway stimulates apoptosis with the increase of caspase-3 in adrenocortical cancer cell line H295R [27] and lung cancer line cells [28]. Inhibition of Notch signaling pathway enhances apoptosis with increasing activated caspase-3 in human tongue carcinoma cells [29] and pancreatic cancer cells [30]. Altogether, these data suggest the mechanistic roles of these molecules during cell proliferation and apoptosis induced by Wnt and Notch signaling pathways. Herein, our observation indicates that the functional link between Wnt and Notch signaling pathways, at least, partly dues to their regulations of cyclinA and caspase-3.

Although there are some differences of the regulations of these two pathways in cell proliferation and apoptosis in the three cell lines, our findings may support the existence of functional link between the two pathways. Further studies are still required to clarify the roles of Wnt and Notch3 signaling pathways in NSCLC. The results of this study would be helpful to understand how interplay between Wnt and Notch3 signaling pathways involved in the carcinogenesis of NSCLC. The double inhibitions of signaling pathways would be an effective strategy for the anti-NSCLC therapy.
